# Pharmaco-multiomics in major depressive disorder: a narrative review and proposed translational framework for difficult-to-treat and treatment-resistant depression

**DOI:** 10.3389/fphar.2026.1934360

**Published:** 2026-09-03

**Authors:** Bernhard T. Baune

**Affiliations:** 1 Department of Psychiatry and Psychotherapy, University of Münster, Münster, Germany; 2 School of Medicine, University of Western Australia, Perth, WA, Australia; 3 Department of Psychiatry, University of Melbourne, Melbourne, VIC, Australia

**Keywords:** difficult-to-treat depression, epigenomics, inflammation, machine learning, major depressive disorder, metabolomics, microbiome, pharmacogenomics

## Abstract

**Background:**

Antidepressant prescribing in major depressive disorder (MDD) remains dominated by sequential trial and error, particularly when illness becomes difficult to treat (DTD) or meets conventional treatment-resistant depression (TRD) criteria.

**Objective:**

This narrative review evaluates pharmacogenomics (PGx), therapeutic drug monitoring (TDM), metabolomics/lipidomics, immune-inflammatory markers, proteomics, transcriptomics, epigenomics, microbiomics, and multi-omics machine learning according to their capacity to improve defined pharmacological decisions.

**Methods:**

Evidence was identified through structured PubMed/MEDLINE searches updated to 29 July 2026, targeted searches of ClinicalTrials.gov and the WHO International Clinical Trials Registry Platform, and backward and forward citation searching. The review was restricted to adult MDD and used author-assigned categories that distinguish prognostic, predictive, pharmacokinetic, and treatment-emergent monitoring markers and differentiate discovery, independent replication, external validation, and clinical utility. The article is a narrative Review, not a PRISMA systematic review; study selection, extraction, and translational classification were performed by one author without formal study-level risk-of-bias grading.

**Results:**

Most evidence derives from general adult MDD rather than prospectively defined DTD/TRD cohorts. Drug-specific, guideline-supported PGx and indication-specific TDM are the most clinically proximal molecular tools, but their value is bounded and depends on medication history, inhibitors and inducers, adherence, organ function, assay coverage, and phenoconversion. Commercial combinatorial PGx trials show small and sometimes nonpersistent clinical effects. Metabolomic, inflammatory, proteomic, transcriptomic, epigenomic, microbiomic, and integrated machine-learning studies identify plausible mechanisms and candidate predictors, but the majority remain discovery-stage or internally validated. Positive subgroup signals, including C-reactive protein-defined differential response, are counterbalanced by null prospective trials and inconsistent thresholds.

**Conclusion:**

Current clinical application should remain limited to systematic medication and interaction review, drug-specific guideline-supported PGx, and indication-specific TDM. Dynamic omics layers should be treated as investigational until locked models are externally validated and prospective biomarker-guided strategies demonstrate incremental decision value, clinical benefit, feasibility, equity, and cost-effectiveness. The review proposes a future-state architecture and staged evidence-to-implementation framework for DTD/TRD.

## 1 Introduction

Major depressive disorder (MDD) is a heterogeneous illness for which initial pharmacological treatment remains largely empirical. Treatment-resistant depression (TRD) is generally operationalized by non-response to two or more adequately delivered antidepressant trials, whereas difficult-to-treat depression (DTD) is a broader longitudinal construct that incorporates persistent symptoms, recurrent relapse, functional burden, comorbidity, treatment intolerance, and patient goals (McAllister-Williams et al., 2020; Rush et al., 2006). These concepts are related but not interchangeable. In this review, adult MDD denotes the evidence base; DTD/TRD denotes the principal clinical application in which the cost of delayed or ineffective decisions is greatest.

Biomarker studies frequently distinguish patients from controls or correlate molecular variation with symptom change. Such findings may advance pathophysiology without improving care. A biomarker becomes treatment-relevant only when it improves a specified decision: which drug to select, how to dose or monitor it, when to switch or augment, how to reduce adverse effects, or when to escalate to advanced pharmacological or neuromodulatory interventions. The relevant comparator is not chance but high-quality clinical assessment, treatment-history reconstruction, measurement-based care, medication and interaction review, and established clinical pharmacology.

Previous work has reviewed omics evidence in TRD and broader pharmaco-multiomics platforms (Amasi-Hartoonian et al., 2022; Dhieb et al., 2025). The present review advances beyond platform-by-platform description in three ways. First, it organizes evidence by the clinical decision to be improved. Second, it asks whether each molecular layer adds value beyond drug-specific PGx and measured exposure. Third, it proposes explicit translational stages and go/no-go criteria that separate mechanistic association from external validation and prospective clinical utility.

Here, pharmaco-multiomics is defined as an integrated precision-psychopharmacology framework that combines true omics layers with non-omics clinical-pharmacology anchors. Germline PGx provides stable information on selected drug-metabolism pathways; TDM provides realized exposure; medication review identifies adherence, dose adequacy, inhibitors, inducers, organ-function constraints, and other causes of phenoconversion. Dynamic omics layers may then characterize metabolic state, inflammation, pathway activity, treatment-emergent adaptation, or drug-microbiome interactions. TDM and single-analyte markers such as C-reactive protein (CRP) are not omics technologies, but they are included because they provide essential comparators and potential anchors for evaluating whether more complex assays add actionable information.

### 1.1 Aim and review questions

The review addresses three questions (McAllister-Williams et al., 2020): what is currently known about PGx and adjacent molecular layers in adult antidepressant response, remission, tolerability, exposure, and resistance (Rush et al., 2006); which findings are prognostic, predictive, pharmacokinetic, or suitable for longitudinal monitoring; and (Amasi-Hartoonian et al., 2022) what evidence is required before a marker or integrated model can support treatment selection in DTD/TRD. The framework deliberately distinguishes evidence generated in general MDD from evidence obtained in explicitly defined DTD/TRD cohorts.

## 2 Methods

### 2.1 Review design

This article is a narrative Review and proposed translational framework. It is not a systematic review and was not conducted or reported as a PRISMA 2020 or PRISMA-S review (Page et al., 2021; Rethlefsen et al., 2021). Accordingly, no PRISMA flow diagram, exhaustive database counts, dual-reviewer screening log, meta-analysis, or formal certainty-of-evidence grading is presented. The structured search and abstraction procedures were used to increase transparency and to support a balanced evidence map rather than to claim exhaustive retrieval.

### 2.2 Population and eligibility

The synthesis was restricted to adults with MDD, including studies that explicitly enrolled TRD, inadequate-response, recurrent, chronic, or DTD-relevant populations. Adolescent and pediatric evidence was outside scope because the identified treatment-evidence base and dosing/guideline context were overwhelmingly adult. Studies in bipolar depression, schizophrenia, healthy volunteers, or mixed diagnostic samples were considered only when adult MDD findings were separately extractable. Table 1 summarizes the scope.

**TABLE 1:** Scope and eligibility criteria for the narrative review.

Domain	Included	Generally excluded
Population	Adults (18 years or older) with MDD, including explicitly defined TRD, inadequate-response, chronic/recurrent, or DTD-relevant cohorts	Pediatric/adolescent-only studies; bipolar depression or schizophrenia without separately extractable MDD data; healthy-control studies without treatment outcomes
Treatment context	Antidepressants, antidepressant combinations, pharmacological augmentation, ketamine/esketamine, anti-inflammatory augmentation, and other pharmacological decisions relevant to MDD.	Psychotherapy-only, lifestyle-only, neuroimaging-only, or animal pharmacology without human treatment-outcome evidence
Marker or model	PGx, TDM/exposure, metabolomics/lipidomics, inflammatory markers, proteomics, transcriptomics, epigenomics, microbiomics, and multi-omics/ML models	Diagnostic-only markers without treatment exposure or outcome; purely mechanistic studies without relevance to response, safety, monitoring, or selection
Outcomes	Response, remission, symptom trajectory, adverse effects, discontinuation, realized exposure, adherence, relapse, treatment resistance, or differential treatment benefit	Cross-sectional case-control classification without a pharmacological outcome
Evidence types	Guidelines, randomized and pragmatic trials, prospective and retrospective cohorts, prediction-model studies, systematic reviews/meta-analyses, and high-value mechanistic studies linked to treatment outcome	Unpublished data, conference abstracts without sufficient methods/results, non-peer-reviewed commercial material, and anecdotal reports

DTD, difficult-to-treat depression; MDD, major depressive disorder; ML, machine learning; PGx, pharmacogenomics; TDM, therapeutic drug monitoring; TRD, treatment-resistant depression. Eligibility and final relevance judgments were made by the author.

### 2.3 Evidence identification

PubMed/MEDLINE was searched from database inception and updated on 29 July 2026. The search used a common adult MDD and pharmacological-treatment block combined with separate domain modules for PGx, TDM/phenoconversion, metabolomics/lipidomics, inflammation, proteomics, transcriptomics, epigenomics, microbiome, and multi-omics/ML. This modular approach was adopted because requiring an omics term in every query would miss clinically relevant TDM and inflammatory studies. ClinicalTrials.gov and the WHO International Clinical Trials Registry Platform were searched using MDD/TRD terms paired with PGx, biomarker, inflammation, metabolomics, proteomics, transcriptomics, microbiome, or precision-treatment terms. Backward references and forward citations of pivotal guidelines, trials, prior reviews, and included studies were checked. No date restriction was applied; English-language full text was required for detailed abstraction. Embase, PsycInfo, Scopus/Web of Science, and CENTRAL were not represented as completed searches.

Table 2 summarizes the search architecture; complete domain-specific PubMed syntax and registry procedures are provided in Supplementary Appendix S3.

**TABLE 2:** Search sources and domain-specific search architecture.

Source/module	Approach
PubMed/MEDLINE: common block	Adult MDD, unipolar depression, TRD, DTD, antidepressants, pharmacotherapy, response, remission, non-response, tolerability, adverse effects, exposure, and treatment outcome
PGx module	pharmacogenomic, pharmacogenetic, CYP2D6, CYP2C19, CYP2B6, gene-drug, combinatorial pharmacogenomic, CPIC, DPWG.
TDM/phenoconversion module	therapeutic drug monitoring, serum/plasma concentration, drug exposure, adherence, phenoconversion, inhibitor, inducer, drug-drug interaction
Dynamic molecular modules	Separate modules for metabolomic/lipidomic; CRP/inflamm/cytokine/interleukin/TNF; proteomic; transcriptomic/gene expression/RNA-seq; epigenomic/methylation; microbiome/microbiota/metagenomic; multi-omics/multiomics/machine learning/prediction model
Registries	ClinicalTrials.gov and WHO ICTRP searches combining depression/MDD/TRD with PGx, biomarker, omics, inflammation, microbiome, or precision-treatment terms
Citation searching	Backward and forward citation searching of pivotal guidelines, randomized trials, systematic reviews, externally validated models, and recent domain-defining studies
Limits and update	Database inception to 29 July 2026; adult human evidence; English full text for detailed abstraction; no date restriction

CPIC, Clinical Pharmacogenetics Implementation Consortium; DTD, difficult-to-treat depression; DPWG, Dutch Pharmacogenetics Working Group; MDD, major depressive disorder; PGx, pharmacogenomics; TDM, therapeutic drug monitoring; TRD, treatment-resistant depression. This is a structured narrative search, not a PRISMA-counted systematic search.

### 2.4 Selection and data abstraction

One author screened titles and abstracts for relevance, assessed potentially eligible full texts, and extracted data into a structured evidence matrix. Extracted fields included population and treatment-resistance status, treatment and comparator, sample size, marker platform and sampling time, outcome definition, quantitative result, validation strategy, intended decision, and principal limitation. When eligibility or interpretation was uncertain, the study was retained for contextual discussion but was not assigned a higher translational category without supporting evidence. Because all screening, extraction, and maturity classification were performed by one author, no duplicate screening, adjudication, or inter-rater reliability estimate was available.

### 2.5 Marker roles and translational maturity

The synthesis separates marker roles that are often conflated. A prognostic marker predicts outcome irrespective of treatment; a predictive marker identifies differential benefit between treatments and therefore requires treatment-by-marker evidence; a pharmacokinetic marker informs metabolism, dose, or exposure; and a treatment-emergent monitoring marker changes after treatment and may indicate target engagement, early response, toxicity, or drift. Translational maturity was assigned by the author using the definitions in Table 3. Internal cross-validation, replication in a second dataset, and external validation of a locked model were not treated as equivalent. Formal study-level tools such as PROBAST and RoB 2 were not applied, although their principles informed the appraisal of model overfitting, calibration, applicability, and randomized-trial limitations (Collins et al., 2015; Wolff et al., 2019; Sterne et al., 2019).

**TABLE 3:** Operational definitions used for marker role and translational maturity.

Term	Definition	Minimum implication
Prognostic marker	Associated with outcome across treatments; does not establish which treatment is preferable	Treatment-independent association or model; may support risk stratification
Predictive marker	Identifies differential benefit or harm between specified treatments	Treatment-by-marker interaction, heterogeneous-treatment-effect model, or prespecified comparative decision rule
Pharmacokinetic marker	Informs metabolism, dose, concentration, exposure, or drug-drug-gene interaction	Drug-specific guideline, exposure-response evidence, or TDM-supported interpretation
Treatment-emergent monitoring marker	Changes during treatment and may indicate target engagement, early response, toxicity, or biological drift	Prespecified longitudinal sampling and temporally appropriate outcome
Discovery/internal validation	Association or model developed and assessed in the same source dataset, including resampling or cross-validation	Hypothesis-generating; high risk of optimism if feature selection and validation are not fully nested
Independent replication	Association confirmed in a separate dataset with the same clinically relevant endpoint	Supports robustness, but a model may still be re-estimated or adapted
External validation	A locked model or rule is applied in an independent cohort, with discrimination and calibration reported when applicable	Required before claiming generalizable predictive performance
Prospective clinical utility	A biomarker-guided strategy improves outcomes versus a strong clinical comparator	Decision-impact or randomized strategy trial with prespecified action rule and patient-relevant outcomes
Guideline-supported selected use	Professional guidance supports a defined drug-marker decision under specified conditions	Not equivalent to universal actionability or endorsement of proprietary multigene algorithms

AUC, area under the receiver-operating characteristic curve; TDM, therapeutic drug monitoring. These author-assigned definitions are informed by prediction-model and risk-of-bias principles (Collins et al., 2015; Wolff et al., 2019; Sterne et al., 2019); they are not the product of a formal consensus or certainty-grading exercise.

### 2.6 Narrative synthesis

Evidence was organized first by clinical-pharmacology anchors and then by dynamic molecular layers. Within each domain, the review asks: what is known; what kind of marker is being studied; whether findings were internally validated, independently replicated, or externally validated; what incremental value was shown beyond clinical variables, PGx, and exposure; and what gap prevents routine use. Quantitative results are provided for pivotal PGx trials, biomarker-enriched anti-inflammatory studies, and externally tested proteomic, transcriptomic, and multi-omics models. Supplementary Table S1 summarizes key studies, and Supplementary Table S2 maps the principal evidence gaps to recommended study designs.

## 3 Results

### 3.1 Evidence base and population framing

The evidence base is uneven. Guideline-supported PGx and TDM draw on drug-specific pharmacokinetic evidence, whereas most dynamic omics studies are smaller observational cohorts or secondary analyses. Critically, much of the evidence derives from general adult MDD populations rather than cohorts meeting uniform DTD/TRD criteria. Evidence was therefore interpreted according to the population actually studied and not automatically generalized to highly resistant illness. Studies explicitly enrolling TRD or inadequate-response populations are identified as such in Supplementary Table S1.

### 3.2 Decision architecture: current anchors and investigational layers

**FIGURE 1:**
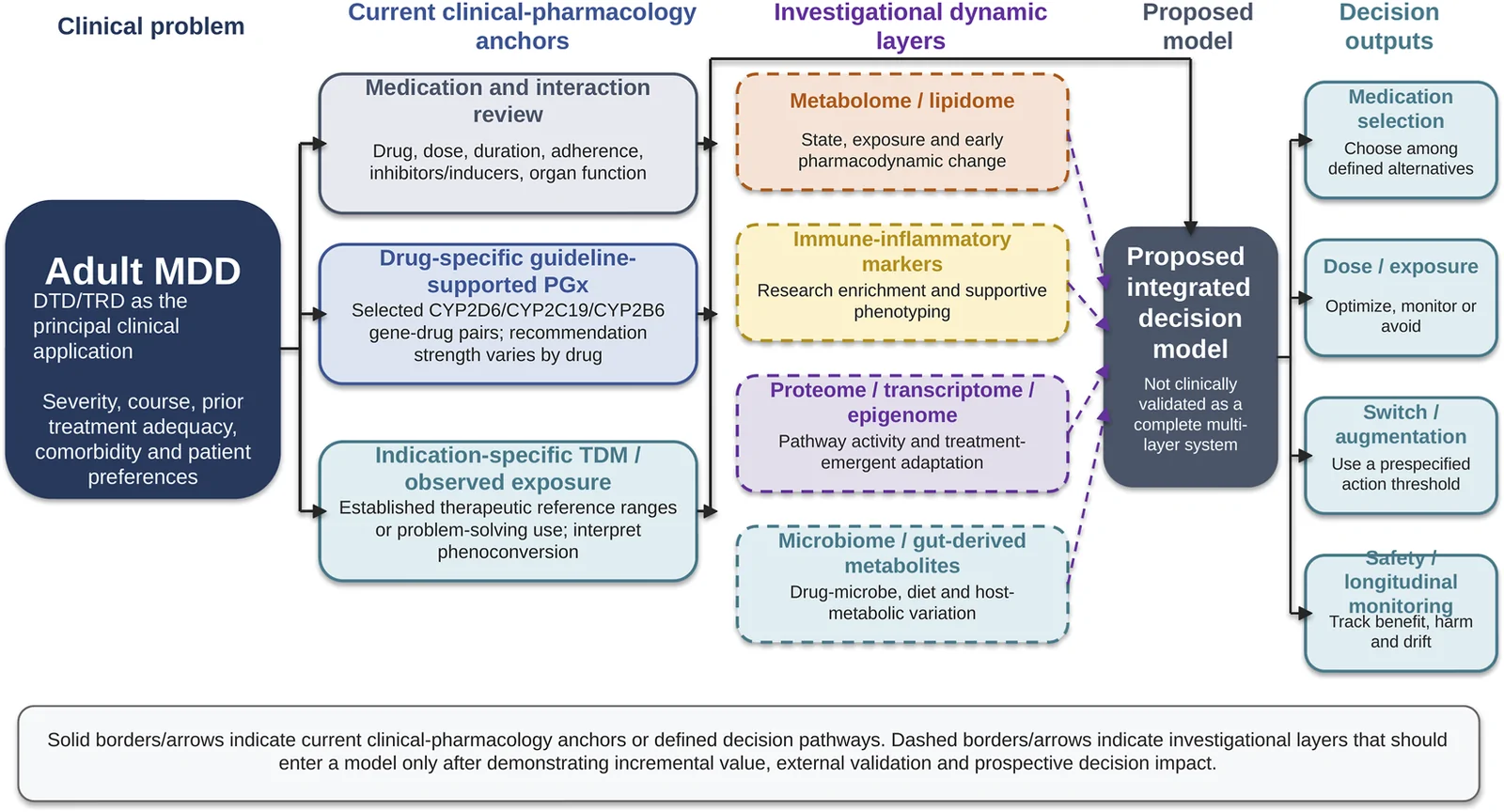
Author-proposed future-state pharmaco-multiomics architecture for adult MDD, with DTD/TRD as the principal clinical application. Solid borders and arrows denote current clinical-pharmacology anchors or defined decision pathways; dashed borders and arrows denote investigational layers. The proposed integrated model is not currently validated as a complete multi-layer clinical system. DTD, difficult-to-treat depression; MDD, major depressive disorder; PGx, pharmacogenomics; TDM, therapeutic drug monitoring; TRD, treatment-resistant depression.

The proposed architecture distinguishes what can already support bounded clinical decisions from what remains investigational. Clinical phenotype, prior treatment adequacy, comorbidity, preferences, medication and interaction review, drug-specific guideline-supported PGx, and indication-specific TDM form the current clinical-pharmacology baseline. Dynamic layers should enter an integrated model only after demonstrating incremental value, external validation, and prospective decision impact. Figure 1 depicts this future-state architecture; it does not imply that a validated complete multi-layer decision model currently exists.

### 3.3 Pharmacogenomics: bounded, drug-specific actionability

PGx is the most mature omics component because selected CYP2D6, CYP2C19, and CYP2B6 gene-drug relationships affect antidepressant metabolism and, for specific drugs and phenotypes, support dose modification or alternative selection. CPIC and DPWG recommendations are drug-specific; the same gene is not uniformly actionable across antidepressants, and recommendation strength varies by substrate, pathway contribution, exposure-response evidence, and therapeutic index (Bousman et al., 2023; Brouwer et al., 2022; Beunk et al., 2024). CPIC does not support SLC6A4 or HTR2A for antidepressant prescribing, so PGx should not be described as providing an established pharmacodynamic prescribing baseline (Bousman et al., 2023).

TCAs are particularly relevant after earlier treatment failures. CPIC guidance addresses CYP2D6 and CYP2C19 for amitriptyline, clomipramine, doxepin, imipramine, trimipramine, nortriptyline, and desipramine, often recommending an alternative, a lower starting dose, or TDM depending on metabolizer status (Hicks et al., 2017). This is conceptually different from evidence for proprietary combinatorial panels, which combine multiple pharmacokinetic and pharmacodynamic variants into vendor-specific algorithms. Clinical validity of an individual gene-drug relationship cannot be assumed to establish the clinical utility of a commercial multigene ranking system.

The largest trials illustrate bounded effects. In GUIDED, 1,167 adults with MDD and inadequate response were randomized to combinatorial PGx-guided care or treatment as usual. At week 8, mean symptom improvement did not differ significantly (27.2% vs. 24.4%; P = 0.107), although response (26.0% vs. 19.9%; P = 0.013) and remission (15.3% vs. 10.1%; P = 0.007) favored guided care (Greden et al., 2019). In PRIME Care, 1,944 adults initiating or switching a single antidepressant were randomized to PGx-guided care or usual care. PGx reduced prescribing of medications with predicted drug-gene interactions and produced a small advantage in remission across 24 weeks (odds ratio 1.28, 95% CI 1.05–1.57; risk difference 2.8%, 95% CI 0.6%–5.1%), but the week-24 difference was not significant (risk difference 1.5%, 95% CI −2.4%–5.3%; P = 0.45) (Oslin et al., 2022). Meta-analyses and umbrella reviews generally suggest modest earlier response or remission advantages, but effects vary with test design, comparator quality, sponsorship, outcome timing, and panel composition (Arnone et al., 2023; Tesfamicael et al., 2024; Sharew et al., 2024).

PGx is also not an error-free baseline. The observed phenotype can diverge from genotype through inhibitors, inducers, inflammation, age, smoking, organ dysfunction, and other causes of phenoconversion (Klomp et al., 2020). In depressed patients, bupropion produced dose-dependent CYP2D6 phenoconversion: 19% at 150 mg/day and 50% at doses of 300 mg/day or greater were phenotypically converted to poor metabolizer status (Hole et al., 2021). Technical coverage matters because CYP2D6 copy-number and structural variation are complex; in 106,474 clinical samples, conversions, deletions, duplications, and multiplications were frequent enough that testing only a limited copy-number site could misclassify phenotype (Frear et al., 2024). PGx results should therefore be interpreted with the medication list, assay coverage, ancestry-relevant alleles, current clinical state, and, when needed, observed exposure.

### 3.4 Medication review, phenoconversion, and TDM

TDM is not an omics technology, but it measures the realized consequence of genotype, adherence, dose, absorption, organ function, interacting drugs, and disease-related phenoconversion. Consensus guidance identifies antidepressants for which therapeutic reference ranges and concentration-related decision support are relatively established, particularly TCAs and selected agents with substantial pharmacokinetic variability. For many other antidepressants, TDM is primarily problem-solving rather than routine: suspected non-adherence, unexpected adverse effects, drug interactions, extreme age, pregnancy, organ dysfunction, unusual dosing, or apparent non-response despite adequate treatment (Hiemke et al., 2018; Piacentino et al., 2022).

The clinically coherent sequence is: genotype-informed phenotype where available; systematic review of medication, dose, duration, adherence, inhibitors, inducers, smoking, inflammation, and organ function; then TDM when the result can resolve an exposure question or guide a dose/action. This sequence is particularly important for CYP2D6 because strong inhibitors can convert a genotypic normal metabolizer into a functional poor metabolizer (Klomp et al., 2020; Hole et al., 2021). A measured concentration should likewise not be interpreted without timing, steady state, active metabolites, sampling quality, and the clinical outcome. PGx and TDM are complementary, not competing, tools.

### 3.5 Metabolomics and lipidomics

Metabolomics and lipidomics can capture dynamic state, exposure, mitochondrial function, amino-acid and one-carbon metabolism, kynurenine biology, acylcarnitines, phospholipids, and sphingolipids. Antidepressant studies have reported baseline or treatment-emergent associations involving acylcarnitines, amines, lipids, and related networks (Joyce et al., 2021; Grant et al., 2022; Costa et al., 2022; Jansen et al., 2024; MahmoudianDehkordi et al., 2021). These signals can illuminate mechanisms and may support early pharmacodynamic monitoring or subtype research.

However, most findings are prognostic or associative rather than comparative treatment predictors. Diet, fasting, body mass index, circadian timing, smoking, comorbidity, sample handling, platform choice, batch effects, and medication exposure can materially change profiles. Few signatures are locked, externally validated, calibrated, or tested as decision rules. Consequently, routine metabolic panels and research-grade metabolomics should not be represented as established antidepressant-selection tools. Their defensible current role is research enrichment, mechanism-focused phenotyping, and prospective evaluation of whether repeated measurements add value beyond clinical trajectory, PGx, and exposure.

### 3.6 Immune-inflammatory markers

CRP and individual cytokines are non-omics markers; multiplex immunophenotyping and pathway-level analyses may approach an omics scale. The literature must distinguish three questions: whether inflammation predicts generally poorer outcome, whether a marker predicts differential benefit between antidepressants, and whether it enriches response to an anti-inflammatory intervention. Meta-analyses support associations between inflammatory signals and antidepressant outcome, but heterogeneity and publication bias remain important (Ga et al., 2022; Liu et al., 2020).

The strongest antidepressant-comparison signal comes from GENDEP. In 241 adults randomized to escitalopram or nortriptyline, baseline CRP interacted with drug (beta = 3.27, 95% CI 1.65–4.89). At CRP below 1 mg/L, escitalopram produced approximately three points more MADRS improvement; at higher CRP, nortriptyline produced approximately three points more improvement. CRP and its interaction with medication explained more than 10% of outcome variance (Uher et al., 2014). This was a treatment-by-marker finding, but it concerned two specific drugs, was not implemented as a prospective decision rule, and requires independent replication. CO-MED analyses also suggested CRP-related differential outcomes across treatment combinations, but did not establish a ready-to-use threshold (Jha et al., 2017).

Biomarker-enriched anti-inflammatory trials provide caution. In a 60-participant infliximab trial in TRD, there was no overall treatment benefit. An exploratory subgroup with baseline hs-CRP above 5 mg/L showed response in 62% (8/13) with infliximab versus 33% (3/9) with placebo (P = 0.19), a hypothesis-generating result rather than confirmatory utility (Raison et al., 2013). MINDEP enrolled 39 patients with TRD and CRP at least 1 mg/L; there was no overall group difference, while an unplanned CRP at least 3 mg/L subgroup contained only six minocycline-treated patients and should be interpreted cautiously (Nettis et al., 2021). In the larger MinoTRD trial, 168 participants were included in the intention-to-treat analysis and adjunctive minocycline did not improve MADRS trajectory versus placebo (mean difference 1.46, 95% CI −1.04 to 3.96; P = 0.25), with no convincing CRP-defined effect (He et al., 2022).

Thresholds used for prognosis, antidepressant comparison, and anti-inflammatory enrichment are therefore not interchangeable. CRP below 1 mg/L in GENDEP, CRP at least 1 or 3 mg/L in MINDEP, and hs-CRP above 5 mg/L in the infliximab trial addressed different treatments and hypotheses. CRP may be useful for research enrichment or supportive phenotyping, but current evidence does not justify routine antidepressant or anti-inflammatory selection on CRP alone.

### 3.7 Proteomics

Proteomic studies identify candidate proteins related to inflammation, endocrine regulation, neuroplasticity, coagulation, and metabolism. Predicted-proteome analyses and treatment-outcome meta-analyses suggest biologically coherent pathways, but many signals rely on genetically inferred protein levels or single cohorts rather than directly measured, independently validated assays (Oliva et al., 2025).

A 2026 serum proteomics study identified sex hormone-binding globulin (SHBG) as a candidate predictor of SSRI remission and reported an AUC of 0.861 after orthogonal verification (Lee et al., 2026). The result is promising but remains a discovery-to-verification finding: the decision threshold, calibration, subgroup performance, incremental value, and independent external validation were not established. SHBG should therefore be considered a candidate marker, not a clinical selection test.

### 3.8 Transcriptomics

Transcriptomics can capture state-dependent pathway activity and treatment-emergent adaptation. Studies implicate immunometabolic, cell-cycle, neuroplasticity, and stress-response programs, but peripheral blood expression is affected by cell composition, infection, circadian timing, smoking, and medication (Sforzini et al., 2025). Early treatment-emergent signatures may be more suitable for monitoring than for pretreatment selection, provided sampling is standardized and temporal ordering is clear.

The impact of external validation is illustrated by a 2026 peripheral-blood model. A random-forest signature achieved an AUC of 0.933 in the discovery dataset but only 0.683 and 0.677 in two independent validation datasets (Kang et al., 2026). The drop is clinically important: high discovery performance did not translate into strong generalizable discrimination, and calibration and prospective utility were not established. Transcriptomic models should report locked feature sets, nested development procedures, external discrimination and calibration, and decision consequences.

### 3.9 Epigenomics

DNA methylation integrates genetic background with environmental and treatment exposures and may therefore capture durable susceptibility and dynamic adaptation. Integrated methylation-expression studies have identified candidate predictors of antidepressant response, while more recent epigenome-wide analyses emphasize heterogeneity and limited replication (Ju et al., 2019; Schiele et al., 2024; Dahrendorff et al., 2024). These markers remain vulnerable to tissue and cell-type composition, smoking, age, trauma, batch effects, and reverse causation. No epigenomic antidepressant-selection rule has established prospective clinical utility.

### 3.10 Microbiomics

Microbiome studies link baseline community structure, microbial functional genes, gut-derived metabolites, diet, and antidepressant exposure. Recent reports include an SSRI treatment cohort, cross-sectional multi-omics analyses, an evidence synthesis of baseline profiles and treatment response, and studies showing that medication exposure itself is associated with microbial features (Jiang et al., 2024; Hernandez-Cacho et al., 2025; Xie et al., 2024; Dilmore et al., 2025). Together, they suggest plausible links to amino-acid and lipid pathways, but apparent prediction is sensitive to geography, diet, sequencing methods, medication history, antibiotics, constipation, and host metabolism.

The microbiome may also mediate drug effects rather than simply predict them, creating bidirectional causality. Current studies are generally small, use heterogeneous endpoints, and rarely validate a locked model across sites. Until standardized collection, functional assays, external validation, and prospective action rules are available, microbiomic findings should remain mechanistic or research-enrichment evidence.

### 3.11 Multi-omics integration and machine learning

Multi-omics models seek to combine stable and dynamic information. In PGRN-AMPS and CO-MED, an integrated genomic-metabolomic model used 264 participants for development and 111 for cross-trial testing, reporting a test AUC of approximately 0.86 and accuracy of 77.5% for acute combination-treatment response (Joyce et al., 2021). This was an important cross-trial replication, but it did not prospectively test whether the model selected a better treatment than high-quality care. Other approaches integrate genetic scores, methylation, expression, inflammation, and clinical variables, but remain limited by high dimensionality, missingness, small effective samples, and instability (Grant et al., 2022; Athreya et al., 2019; Fuh et al., 2023; Yadav et al., 2025).

A central conceptual gap is the difference between predicting response and selecting treatment. A model that predicts poor outcome across all options is prognostic. Treatment selection requires estimates of differential benefit between alternatives, ideally through prespecified treatment-by-feature interactions, counterfactual or individual-treatment-effect methods, calibrated medication-specific predictions, and prospective testing of an interpretable decision rule. Recent reviews show that external validation and calibration are uncommon and that only a minority of models directly rank treatments for an individual patient (Arnold et al., 2024; He et al., 2026). Algorithmic complexity cannot substitute for comparative design.

### 3.12 Evidence map and current clinical positioning

Table 4 integrates marker role, representative evidence, validation status, and current clinical positioning while maintaining a clear distinction between drug-specific guideline-supported PGx and proprietary combinatorial PGx panels, because these represent different interventions with different evidentiary bases. Selected clinical use is reserved for defined gene-drug recommendations and indication-specific TDM; proprietary combinatorial panels are summarized separately according to their trial evidence and limitations.

**TABLE 4:** Decision-oriented evidence map and current positioning of pharmaco-multiomics layers.

Layer	Primary role	Representative evidence	Evidence status	Current position
Medication and interaction review	Clinical/pharmacological anchor	Dose, duration, adherence, inhibitors/inducers, organ function, prior treatment adequacy	Established standard of care; no molecular validation category	Use now for every patient; forms the comparator for any biomarker strategy
Drug-specific guideline-supported PGx	Pharmacokinetic; occasionally safety/exposure	CPIC/DPWG recommendations for selected CYP2D6, CYP2C19, and CYP2B6 gene-drug pairs, including drug-specific TCA recommendations (Bousman et al., 2023; Brouwer et al., 2022; Beunk et al., 2024; Hicks et al., 2017)	Guideline-supported for selected, explicitly defined gene-drug pairs; recommendation strength varies by drug and phenotype	Use selectively for defined gene-drug pairs; interpret with assay coverage, medication interactions, phenoconversion, ancestry-relevant variation, and observed exposure where appropriate
Proprietary combinatorial PGx panels	Vendor-specific multigene medication-ranking or decision-support intervention	GUIDED and PRIME Care randomized trials and related meta-analyses/umbrella reviews, showing small, bounded, and in some analyses nonpersistent clinical effects (Greden et al., 2019; Oslin et al., 2022; Arnone et al., 2023; Tesfamicael et al., 2024; Sharew et al., 2024)	Randomized-trial evidence exists, but algorithms, gene content, medication categories, comparator quality, sponsorship, and outcome timing are heterogeneous; this evidence is distinct from CPIC/DPWG drug-gene guidance	Do not present as equivalent to guideline-supported drug-specific PGx. Interpret as a separate commercial decision-support strategy whose generalizability and incremental utility depend on the specific panel and clinical context
TDM/observed exposure	Pharmacokinetic and monitoring	Consensus reference ranges and problem-solving indications (Hiemke et al., 2018; Piacentino et al., 2022)	Clinical use for selected drugs/situations; not universal	Use when a concentration can resolve adherence, interaction, exposure, toxicity, or dose questions
Metabolomics/lipidomics	Mostly prognostic or treatment-emergent monitoring	Acylcarnitine, amine, lipid and network signals; cross-trial multi-omics prediction (Joyce et al., 2021; Grant et al., 2022; Costa et al., 2022; Jansen et al., 2024; MahmoudianDehkordi et al., 2021)	Discovery/replication; sparse locked external validation and utility	Research enrichment and mechanistic monitoring; not routine treatment selection
Immune-inflammatory markers	Prognostic; candidate predictive/enrichment	GENDEP CRP interaction; CO-MED secondary analysis; null overall infliximab, MINDEP and MinoTRD results (Jha et al., 2017; Raison et al., 2013; Nettis et al., 2021; Ga et al., 2022; Liu et al., 2020; Uher et al., 2014; He et al., 2022)	Mixed; treatment- and threshold-specific; no prospectively validated general rule	Research enrichment/supportive phenotyping only; do not direct routine antidepressant or anti-inflammatory selection
Proteomics	Candidate prognostic/predictive	Predicted-proteome studies and SHBG candidate AUC 0.861 (23,47)	Discovery/verification; no independent external clinical validation	Research only
Transcriptomics	Prognostic or treatment-emergent monitoring	Immunometabolic pathways; AUC 0.933 discovery and 0.683/0.677 validation (Sforzini et al., 2025; Kang et al., 2026)	External performance modest; calibration/utility absent	Research only; prioritize locked multi-site validation
Epigenomics	Candidate prognostic and longitudinal	Methylation-expression and EWAS findings (Ju et al., 2019; Schiele et al., 2024; Dahrendorff et al., 2024)	Discovery/limited replication; strong confounding and tissue effects	Research only
Microbiomics	Candidate prognostic, mechanistic, treatment-emergent	Baseline and functional signatures linked to SSRI outcomes (Jiang et al., 2024; Hernandez-Cacho et al., 2025; Xie et al., 2024; Dilmore et al., 2025)	Mostly small, site-dependent, internally validated	Research enrichment/mechanistic study only
Multi-omics/ML	Prognostic or comparative prediction, depending design	Cross-trial models and integrative signatures (Joyce et al., 2021; Grant et al., 2022; Athreya et al., 2019; Fuh et al., 2023; Yadav et al., 2025; Arnold et al., 2024; He et al., 2026)	Few locked external validations; comparative decision models rare; utility absent	Research only until calibrated comparative models improve prospective decisions

AUC, area under the receiver-operating characteristic curve; CPIC, Clinical Pharmacogenetics Implementation Consortium; DPWG, Dutch Pharmacogenetics Working Group; ML, machine learning; PGx, pharmacogenomics; TDM, therapeutic drug monitoring. Evidence-status assignments and current-position statements are the author’s appraisal, not a formal consensus or certainty grade. “Selected clinical use” requires a defined drug-marker decision supported by guideline or exposure evidence; it does not denote universal actionability.

### 3.13 Author-proposed evidence-to-implementation roadmap

**FIGURE 2:**
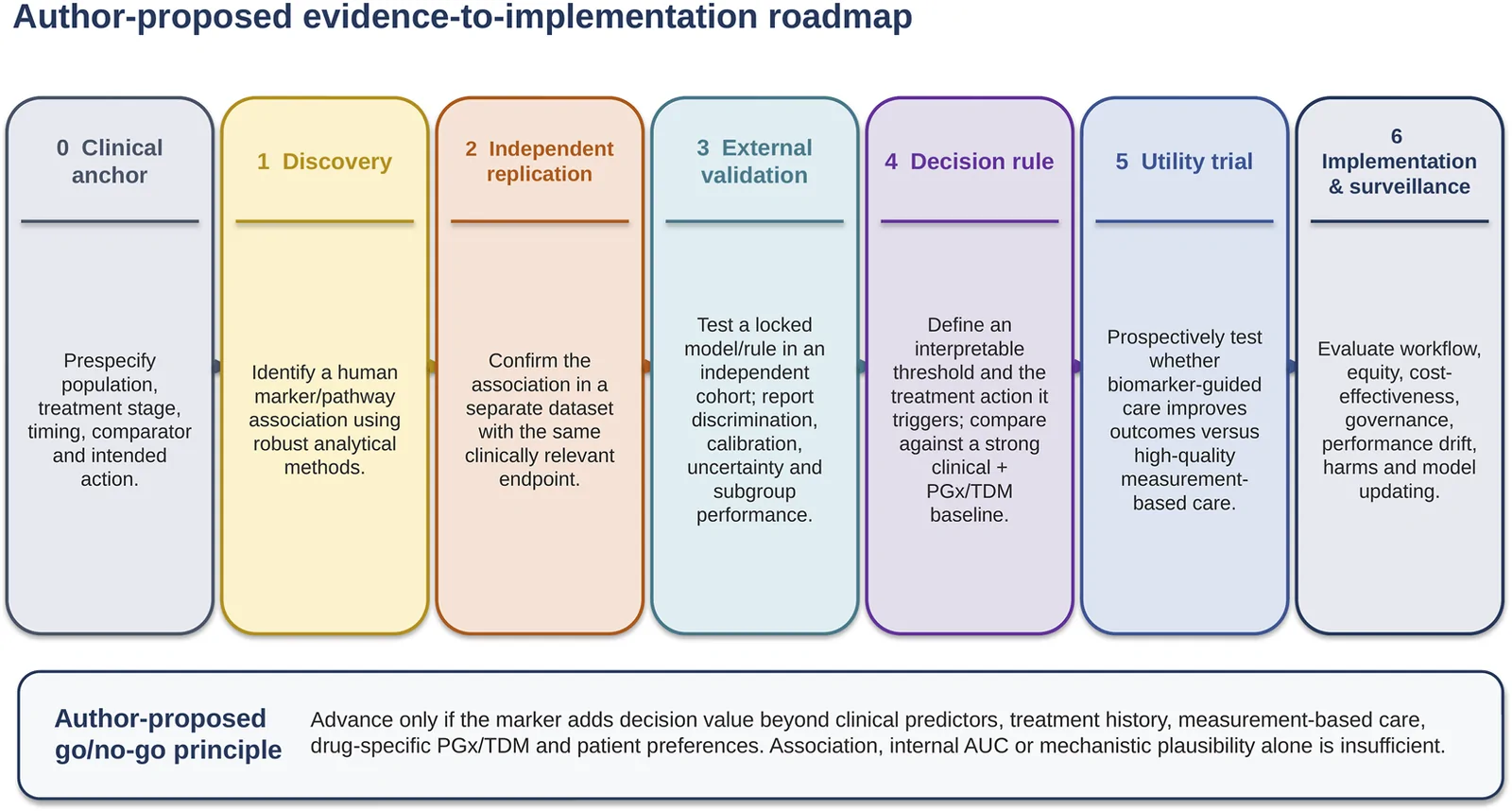
Author-proposed evidence-to-implementation roadmap for pharmaco-multiomics. The stages are cumulative: a clinically anchored marker proceeds from discovery to independent replication, external validation of a locked model or rule, definition of an interpretable action threshold, prospective testing of biomarker-guided care, and implementation with equity, cost-effectiveness, governance, harm monitoring, and model updating. The roadmap is an author-proposed framework, not a formal consensus or systematic certainty-grading scheme. AUC, area under the receiver-operating characteristic curve; PGx, pharmacogenomics; TDM, therapeutic drug monitoring.

The field needs a staged sequence that prevents discovery performance from being interpreted as clinical readiness. Figure 2 presents an author-proposed roadmap from a prespecified clinical anchor through discovery, independent replication, external validation, an interpretable decision rule, a prospective utility trial, and implementation with surveillance. Advancement should stop when a marker fails to add decision value beyond a strong clinical, PGx, and TDM baseline.

### 3.14 Principal evidence gaps

Across domains, recurrent gaps include inconsistent DTD/TRD definitions, underpowered high-dimensional discovery, incomplete pre-analytic reporting, failure to distinguish prognostic from predictive effects, optimistic internal validation, limited calibration and subgroup assessment, absent comparative-treatment modeling, and few prospective decision-impact trials. Supplementary Table S2 links these gaps to recommended designs. The central requirement is not another retrospective classifier, but a study that tests whether a defined test-and-treatment strategy improves patient-relevant outcomes over strong measurement-based care.

## 4 Discussion

Four conclusions emerge. First, current molecular clinical practice is narrower than the phrase pharmaco-multiomics may imply. Drug-specific guideline-supported PGx and indication-specific TDM can improve selected pharmacokinetic decisions, but neither predicts antidepressant efficacy comprehensively. Their value depends on medication history, interaction review, adherence, phenotype conversion, assay quality, and the precise drug-gene or concentration question.

Second, biological informativeness is not equivalent to decision utility. Metabolic, inflammatory, proteomic, transcriptomic, epigenomic, and microbiomic studies repeatedly identify plausible pathways and candidate markers, but many findings are prognostic, internally validated, or treatment-emergent rather than comparative predictors. The inflammatory literature is especially instructive: a compelling CRP-by-drug interaction and exploratory biomarker-enriched signals coexist with null overall prospective trials and noninterchangeable thresholds. These markers should not be combined routinely into treatment-selection panels before replication and prospective utility testing.

Third, external validation and comparative design are the principal translational bottlenecks. High AUCs in discovery datasets commonly attenuate in independent cohorts. A clinically useful selection model must compare alternatives and estimate differential benefit, not simply classify likely responders. Reporting should include calibration, uncertainty, missing-data handling, subgroup performance, and decision-curve or net-benefit analyses. A model should be locked before external testing, and the complete action rule should be evaluated prospectively.

Fourth, the proposed architecture is intentionally hierarchical. Clinical assessment and treatment-history reconstruction remain foundational. PGx and TDM should be used when the decision is drug-specific and interpretable. Dynamic layers should be added only when they improve an explicit decision beyond that baseline. This preserves a plausible route to multi-omics without allowing molecular complexity to outrun evidence.

### 4.1 Clinical implications

For current practice, the defensible sequence is: reconstruct diagnosis, course, prior-treatment adequacy, adherence, adverse effects, and preferences; review all medications, inhibitors, inducers, smoking, organ function, and other causes of phenoconversion; use guideline-supported PGx for relevant drug-gene pairs; and use TDM when an exposure result can change dose, interpret non-response or toxicity, or clarify adherence/interactions. Inflammatory, metabolic, and other dynamic assays may support research recruitment or descriptive phenotyping but should not be presented as validated stand-alone prescribing tests.

### 4.2 Limitations

This narrative review is not exhaustive. It did not include completed searches of every subscription database, PRISMA study counts, duplicate screening, duplicate data extraction, formal risk-of-bias scoring, or quantitative synthesis. Study selection, evidence abstraction, and translational-maturity classification were performed by one author. The evidence base itself is heterogeneous in population, treatment, assay, sampling, outcome, and validation design, and much of it comes from general adult MDD rather than prospectively defined DTD/TRD. Formal tools such as PROBAST and RoB 2 were not applied at study level (Wolff et al., 2019; Sterne et al., 2019). The evidence map and roadmap should therefore be read as an evidence-informed, author-proposed translational synthesis rather than a consensus guideline or certainty-graded systematic review.

### 4.3 Ethics, implementation, and equity

Precision tools can amplify inequity if assays underrepresent ancestry-specific variation, models are trained in narrow populations, or costly testing is restricted to well-resourced services. Microbiome and metabolomic signatures may encode diet, geography, socioeconomic status, and access as much as intrinsic biology. Implementation should include transparent consent, communication of uncertainty, avoidance of deterministic language, subgroup calibration, accessibility, clinician education, electronic-health-record integration, data governance, and ongoing audit for drift and harm. Fairness and cost-effectiveness are components of clinical utility, not *post hoc* additions.

### 4.4 Author-proposed minimum evidence package

Table 5 translates the roadmap into an author-proposed minimum evidence package. It is cumulative rather than a menu: a marker should not progress because it performs well on one criterion while failing analytical validity, external validation, or clinical utility. The first requirement is a precise decision anchor—the adult population, treatment stage, sampling time, available alternatives, action threshold, and intended action. This separates a generally prognostic association from a predictive or pharmacokinetic decision tool.

**TABLE 5:** Author-proposed minimum evidence package before clinical implementation of a pharmaco-multiomics marker or model.

Requirement	Operational criterion	Minimum evidence
1. Clinical decision anchor	Prespecify adult population, treatment stage, timing, comparator options, action threshold, and intended action	Protocol and analysis plan define whether the marker addresses selection, dose/exposure, switch/augmentation, safety, or monitoring
2. Analytical validity	Demonstrate reproducibility, pre-analytic standards, platform stability, QC thresholds, and clinically compatible turnaround	Inter- and intra-assay precision, missingness, batch correction, sample stability, and failure rates reported
3. Clinical validity	Show a clinically meaningful association in an appropriate treatment context	Effect estimate and uncertainty; distinction between prognostic, predictive, pharmacokinetic, and monitoring roles
4. Independent replication and external validation	Replicate the signal and test a locked model/rule in independent cohorts	Discrimination, calibration, uncertainty, subgroup performance, and transportability reported (Collins et al., 2015; Wolff et al., 2019)
5. Incremental and comparative value	Demonstrate added value beyond clinical predictors, treatment history, medication/interactions review, PGx, and TDM.	Nested model comparison, net benefit, actionable reclassification, and treatment-by-marker or heterogeneous-treatment-effect evidence for selection
6. Prospective clinical utility	Test whether biomarker-guided care improves outcomes versus strong measurement-based care	Prespecified decision rule; response/remission, time to effective treatment, harms, function, patient-reported outcomes, and discontinuation
7. Implementation, economics, and equity	Establish workflow feasibility, interpretability, access, acceptability, governance, and cost-effectiveness	Turnaround, EHR integration, clinician action, patient uptake, ancestry/sex/age/comorbidity calibration, reimbursement, and fairness
8. Learning-system surveillance	Monitor performance after deployment	Drift, changing prescribing, inequitable access, adverse consequences, model updates, version control, and withdrawal criteria

AUC, area under the receiver-operating characteristic curve; EHR, electronic health record; PGx, pharmacogenomics; QC, quality control; TDM, therapeutic drug monitoring. This table is the author’s proposed framework informed by PGx/TDM, guidance and prediction-model principles (Bousman et al., 2023; Brouwer et al., 2022; Beunk et al., 2024; Hiemke et al., 2018; Collins et al., 2015; Wolff et al., 2019; Sterne et al., 2019; Hicks et al., 2017); it is not a formal consensus, guideline, or certainty-grading instrument.

Analytical and clinical validity are distinct. Dynamic assays require standardized sampling, pre-analytic handling, platform quality control, batch management, and turnaround time. Clinical validity then requires independent replication and external validation of a locked rule with discrimination, calibration, uncertainty, missing-data handling, and subgroup performance. For treatment selection, evidence must address differential benefit between alternatives rather than response under a single treatment.

Incremental value is the defining pharmaco-multiomics test. A new layer should improve prediction, calibration, actionable reclassification, decision-curve net benefit, or time to effective treatment beyond a strong baseline that includes clinical variables, treatment history, medication/interactions review, drug-specific PGx, and TDM when indicated. Statistical significance or a small internally estimated increase in AUC is insufficient. Prospective clinical utility must evaluate the complete test-and-treatment strategy: assay, interpretation, action rule, clinician behavior, patient uptake, outcomes, adverse effects, functioning, cost, and equity.

The package can be used developmentally: discovery studies should state which criterion they address and what evidence would justify progression. For commercial or service-level deployment, however, absence of independent external validation, a prospective decision-impact test, or equity/implementation evidence should preclude claims of routine readiness. Post-deployment surveillance is essential because prescribing patterns, patient populations, assays, and models change over time.

## 5 Conclusion

Pharmaco-multiomics in MDD should be developed as a decision-led extension of clinical pharmacology, with DTD/TRD as a high-priority application. The current clinical route is limited to systematic medication and interaction review, drug-specific guideline-supported PGx, and indication-specific TDM. Metabolomic, inflammatory, proteomic, transcriptomic, epigenomic, microbiomic, and multi-omics/ML layers remain investigational. Progress requires prespecified comparative decisions, independent external validation of locked models, and prospective trials showing that biomarker-guided strategies improve outcomes, safety, equity, and value beyond high-quality clinical care.
