# Supplementary material for: Pharmaco-multiomics in major depressive disorder: a narrative review and proposed translational framework for difficult-to-treat and treatment-resistant depression
Source: Front Pharmacol. 2026 Sep 3;17:1934360. doi: 10.3389/fphar.2026.1934360 (PMC13583672; doi:10.3389/fphar.2026.1934360)
Supplement: Supplementary file 1 [file Supplementaryfile1.docx]

**Supplementary Material**

Pharmaco-Multiomics in Major Depressive Disorder: A Narrative Review and Proposed Translational Framework for Difficult-to-Treat and Treatment-Resistant Depression

Bernhard T. Baune

# Supplementary Table S1. Key studies informing the narrative evidence map

| **Domain / citation** | **Population / N** | **Treatment / comparator** | **Marker / timing** | **Key quantitative result** | **Validation status** | **Principal limitation** |
| --- | --- | --- | --- | --- | --- | --- |
| Combinatorial PGx; GUIDED (10) | Adult MDD with inadequate response; N=1,167 | PGx-guided care vs usual care | Commercial multigene test; pretreatment | Week-8 symptom improvement 27.2% vs 24.4% (P=.107); response 26.0% vs 19.9% (P=.013); remission 15.3% vs 10.1% (P=.007). | Randomized strategy trial | Primary symptom-improvement endpoint negative; proprietary algorithm; DTD/TRD definition not uniform. |
| PGx; PRIME Care (11) | Adult MDD initiating/switching antidepressant; N=1,944 | PGx-guided care vs usual care | Commercial drug-gene interaction test; pretreatment | Lower DGI prescribing; remission across 24 weeks OR 1.28 (95% CI 1.05-1.57), RD 2.8%; week-24 RD 1.5% (95% CI -2.4 to 5.3). | Large pragmatic randomized trial | Small, nonpersistent clinical effect; mostly general MDD. |
| PGx-metabolomics ML; Joyce et al. (15) | PGRN-AMPS N=264; CO-MED N=111 | Combination antidepressant response | Baseline genomics + metabolomics | Cross-trial test AUC about 0.86; accuracy 77.5%. | Cross-trial replication | Not a prospective comparative treatment-selection trial; calibration/utility not established. |
| Metabolomics; MahmoudianDehkordi et al. (37) | Adult MDD treated with citalopram/escitalopram | SSRI treatment | Acylcarnitines, amines, lipids; baseline/longitudinal | Treatment-related metabolic changes implicated mitochondrial and lipid pathways. | Discovery/replication across samples | Dynamic confounding; no locked decision rule. |
| CRP differential prediction; GENDEP (45) | Adult MDD; N=241 | Escitalopram vs nortriptyline | Baseline hs-CRP | CRP-by-drug beta 3.27 (95% CI 1.65-4.89); approximately 3 MADRS points differential; >10% variance explained. | Randomized treatment-by-marker secondary analysis | Two specific drugs; no independent prospective decision-rule validation. |
| CRP; CO-MED (19) | Adult MDD outpatient sample | Escitalopram, bupropion-escitalopram, venlafaxine-mirtazapine | Baseline CRP | CRP associated with differential outcomes across regimens. | Secondary comparative analysis | Post hoc thresholds and multiple comparisons; utility untested. |
| Infliximab; Raison et al. (20) | TRD; N=60 | Infliximab vs placebo | Baseline hs-CRP and inflammatory markers | No overall benefit; hs-CRP >5 mg/L subgroup response 62% (8/13) vs 33% (3/9), P=.19. | Randomized proof-of-concept; exploratory subgroup | Subgroup small and not statistically confirmatory. |
| Minocycline; MINDEP (21) | TRD with CRP >=1 mg/L; N=39 | Minocycline vs placebo adjunct | Baseline CRP | No overall group difference; CRP >=3 mg/L subgroup signal based on 6 minocycline-treated patients. | Randomized biomarker-enriched trial; exploratory subgroup | Underpowered; subgroup not prespecified; threshold not validated. |
| Minocycline; MinoTRD (46) | TRD; ITT N=168 | Minocycline vs placebo adjunct | Clinical/inflammatory analyses | MADRS mean difference 1.46 (95% CI -1.04 to 3.96), P=.25; no convincing biomarker-defined efficacy. | Multicenter randomized trial | Negative primary and secondary outcomes. |
| Proteomics; Lee et al. (47) | Adult MDD receiving SSRIs | SSRI remission vs non-remission | Serum proteomics; baseline | SHBG candidate; AUC 0.861 after orthogonal verification. | Discovery plus orthogonal verification | No independent external validation, calibration, or utility study. |
| Transcriptomics; Kang et al. (40) | Adult MDD datasets | Antidepressant response | Peripheral blood expression; baseline | AUC 0.933 discovery; 0.683 and 0.677 in independent validation datasets. | Independent external datasets | Performance attenuation; calibration and clinical utility absent. |
| Transcriptomics; Sforzini et al. (22) | Adult MDD with differing inflammation | Antidepressant treatment | Peripheral transcriptome | Immunometabolic and cell-cycle pathways differed by inflammatory state. | Mechanistic/observational | Not a locked comparative selection model. |
| Epigenomics; Ju et al. (24) | Adult MDD cohorts | Antidepressant response | Methylation + expression | Integrated signatures associated with response. | Discovery/integrative validation | Tissue/cell composition and replication limitations. |
| Epigenomics; Schiele et al. (25) | Adult unipolar depression | Antidepressant response | EWAS methylation | Candidate predictive methylation signals. | Discovery with internal assessment | No prospective decision rule or utility. |
| Microbiome-metabolome; Jiang et al. (27) | Adult MDD | SSRI treatment | Gut microbiota + metabolites | Baseline/longitudinal pathways associated with SSRI efficacy. | Observational multi-omics | Diet, geography, medication and platform confounding; external validation limited. |
| Microbiome evidence synthesis; Xie et al. (29) | Adult depression studies | Antidepressant treatment | Baseline gut microbiota | Review summarized associations between baseline microbial profiles and remission/non-remission across small heterogeneous studies. | Narrative evidence synthesis | No new cohort or locked predictor; constituent studies are small and methodologically heterogeneous. |
| Multi-omics; Fuh et al. (41) | Adult MDD | Antidepressant response | Genotype, methylation, expression | Integrated meta-phenotypes implicated immune/inflammatory pathways. | Internal modeling | High-dimensional model; external calibration/utility absent. |
| Comparative ML review; He et al. (44) | Adult MDD studies across >=2 treatments | Comparative pharmacological selection | ML models | Only 7 studies externally validated; most did not distinguish prognostic from predictive markers; one unified patient-level ranking framework. | Systematic review | Field remains early; calibration, explainability and utility largely absent. |

**Note:** AUC, area under the receiver-operating characteristic curve; CRP, C-reactive protein; DGI, drug-gene interaction; DTD, difficult-to-treat depression; EWAS, epigenome-wide association study; hs-CRP, high-sensitivity CRP; ITT, intention to treat; MADRS, Montgomery-Asberg Depression Rating Scale; MDD, major depressive disorder; ML, machine learning; OR, odds ratio; PGx, pharmacogenomics; RD, risk difference; SHBG, sex hormone-binding globulin; SSRI, selective serotonin reuptake inhibitor; TRD, treatment-resistant depression. Evidence-status labels are author-assigned. Reference numbers correspond to the main manuscript.

# Supplementary Table S2. Evidence gaps and recommended study designs

| **Gap** | **Why it matters** | **Recommended design** |
| --- | --- | --- |
| Nonuniform DTD/TRD definitions | Biomarker effects may depend on resistance stage, chronicity, adherence, and prior-treatment adequacy. | Use explicit consensus definitions; record drug, dose, duration, adherence, exposure, intolerance, and outcome for every prior trial. |
| Prognostic-predictive conflation | General response prediction cannot select between treatments. | Randomized or comparative cohorts with treatment-by-marker interactions, heterogeneous-treatment-effect models, and calibrated treatment-specific predictions. |
| Optimistic internal validation | High-dimensional feature selection creates exaggerated AUCs. | Fully nested development; locked models; independent multi-site external validation with calibration and uncertainty. |
| Weak baseline comparators | Complex omics may recapitulate clinical history or exposure. | Compare clinical-only, clinical+medication review, clinical+PGx, clinical+PGx+TDM, and added-omics models. |
| Pre-analytic heterogeneity | Dynamic assays vary with fasting, time, infection, smoking, storage, batch, and cell composition. | Standard operating procedures, repeated samples, QC thresholds, platform harmonization, and blinded assay analysis. |
| Few decision-impact trials | Association and AUC do not establish better care. | Randomized biomarker-guided strategy trials with prespecified action thresholds, time-to-effective-treatment, harms, function, patient outcomes, and cost. |
| Limited diversity and equity assessment | Models may fail across ancestry, sex, age, comorbidity, geography, or socioeconomic context. | Diverse recruitment, subgroup calibration, fairness audits, transportability analyses, access and reimbursement evaluation. |
| No post-deployment surveillance | Model performance and clinical behavior change over time. | Prospective registries, drift and harm monitoring, version control, retraining governance, and withdrawal criteria. |

**Note:** AUC, area under the receiver-operating characteristic curve; DTD, difficult-to-treat depression; PGx, pharmacogenomics; QC, quality control; TDM, therapeutic drug monitoring; TRD, treatment-resistant depression. This is an author-proposed research agenda.

# Supplementary Appendix S3. Search architecture and procedures

Search date and sources. PubMed/MEDLINE was searched from inception and updated on 29 July 2026. Targeted registry searches were conducted in ClinicalTrials.gov and the WHO International Clinical Trials Registry Platform. Backward and forward citation searching was performed from pivotal guidelines, randomized trials, prior reviews, and key included studies. Searches were restricted to human adult MDD evidence for detailed abstraction; English full text was required. No date limit was applied. Embase, PsycInfo, Scopus/Web of Science, and CENTRAL were not represented as completed searches.

Common PubMed population/treatment block:

(("Depressive Disorder, Major"[Mesh] OR depress*[tiab] OR "major depressive disorder"[tiab] OR MDD[tiab] OR "unipolar depression"[tiab] OR "treatment-resistant depression"[tiab] OR "treatment resistant depression"[tiab] OR TRD[tiab] OR "difficult-to-treat depression"[tiab] OR DTD[tiab]) AND (antidepress*[tiab] OR psychopharmacolog*[tiab] OR pharmacotherap*[tiab] OR SSRI*[tiab] OR SNRI*[tiab] OR ketamine[tiab] OR esketamine[tiab] OR augmentation[tiab] OR "drug therapy"[Subheading]) AND (response[tiab] OR remission[tiab] OR nonresponse[tiab] OR "non-response"[tiab] OR tolerability[tiab] OR "adverse effect*"[tiab] OR "treatment outcome"[tiab] OR exposure[tiab] OR monitoring[tiab]))

**PGx module:**

(pharmacogenomic*[tiab] OR pharmacogenetic*[tiab] OR CYP2D6[tiab] OR CYP2C19[tiab] OR CYP2B6[tiab] OR "gene-drug"[tiab] OR "combinatorial pharmacogenomic*"[tiab] OR CPIC[tiab] OR DPWG[tiab])

**TDM/phenoconversion module:**

("therapeutic drug monitoring"[tiab] OR TDM[tiab] OR "drug concentration"[tiab] OR "serum concentration"[tiab] OR "plasma concentration"[tiab] OR phenoconversion[tiab] OR inhibitor*[tiab] OR inducer*[tiab] OR "drug-drug interaction*"[tiab])

**Metabolomics/lipidomics module:**

(pharmacometabolomic*[tiab] OR metabolomic*[tiab] OR lipidomic*[tiab] OR acylcarnitine*[tiab] OR sphingomyelin*[tiab] OR phospholipid*[tiab])

**Inflammation module:**

("C-reactive protein"[tiab] OR CRP[tiab] OR inflamm*[tiab] OR cytokine*[tiab] OR interleukin*[tiab] OR "tumor necrosis factor"[tiab] OR TNF[tiab] OR immunophenotyp*[tiab])

**Proteomics module:**

(proteomic*[tiab] OR "mass spectrometry"[tiab] OR "protein signature"[tiab] OR "plasma protein*"[tiab] OR "serum protein*"[tiab])

**Transcriptomics module:**

(transcriptomic*[tiab] OR "gene expression"[tiab] OR RNA-seq[tiab] OR RNAseq[tiab] OR "expression signature"[tiab])

**Epigenomics module:**

(epigenomic*[tiab] OR methylation[tiab] OR "DNA methylation"[tiab] OR EWAS[tiab])

**Microbiome module:**

(microbiome[tiab] OR microbiota[tiab] OR metagenomic*[tiab] OR "gut-derived metabolite*"[tiab] OR "drug-microbe"[tiab])

**Multi-omics/ML module:**

("multi-omics"[tiab] OR multiomics[tiab] OR "machine learning"[tiab] OR "prediction model"[tiab] OR "treatment selection"[tiab] OR "heterogeneous treatment effect*"[tiab] OR counterfactual[tiab])

Each domain query combined the common block with the corresponding module using AND. Searches were supplemented with domain-specific author, study-name, guideline, and citation queries when pivotal evidence was identified. Registry searches used simplified combinations of depression/MDD/TRD with pharmacogenomic, biomarker, inflammation, metabolomic, proteomic, transcriptomic, microbiome, multi-omics, or precision-treatment terms. Records were selected and abstracted by one author; no duplicate screening or formal PRISMA accounting was performed.
